# Serum-Induced Differentiation of Glioblastoma Neurospheres Leads to Enhanced Migration/Invasion Capacity That Is Associated with Increased MMP9

**DOI:** 10.1371/journal.pone.0145393

**Published:** 2015-12-23

**Authors:** Justin V. Joseph, Ingrid A. M. van Roosmalen, Ellen Busschers, Tushar Tomar, Siobhan Conroy, Ellie Eggens-Meijer, Natalia Peñaranda Fajardo, Milind M. Pore, Veerakumar Balasubramanyian, Michiel Wagemakers, Sjef Copray, Wilfred F. A. den Dunnen, Frank A. E. Kruyt

**Affiliations:** 1 Department of Medical Oncology, University of Groningen, University Medical Center Groningen, Groningen, The Netherlands; 2 Department of Pharmacy, University of Groningen, University Medical Center Groningen, Groningen, The Netherlands; 3 Department of Gynecologic Oncology, University of Groningen, University Medical Center Groningen, Groningen, The Netherlands; 4 Department of Pathology, University of Groningen, University Medical Center Groningen, Groningen, The Netherlands; 5 Department of Neuroscience, University of Groningen, University Medical Center Groningen, Groningen, The Netherlands; 6 Department of Neuro-surgery, University of Groningen, University Medical Center Groningen, Groningen, The Netherlands; University of Florida, UNITED STATES

## Abstract

Glioblastoma (GBM) is a highly infiltrative brain tumor in which cells with properties of stem cells, called glioblastoma stem cells (GSCs), have been identified. In general, the dominant view is that GSCs are responsible for the initiation, progression, invasion and recurrence of this tumor. In this study, we addressed the question whether the differentiation status of GBM cells is associated with their invasive capacity. For this, several primary GBM cell lines were used, cultured either as neurospheres known to enrich for GSCs or in medium supplemented with 10% FCS that promotes differentiation. The differentiation state of the cells was confirmed by determining the expression of stem cell and differentiation markers. The migration/invasion potential of these cells was tested using *in vitro* assays and intracranial mouse models. Interestingly, we found that serum-induced differentiation enhanced the invasive potential of GBM cells, which was associated with enhanced MMP9 expression. Chemical inhibition of MMP9 significantly reduced the invasive potential of differentiated cells *in vitro*. Furthermore, the serum-differentiated cells could revert back to an undifferentiated/stem cell state that were able to form neurospheres, although with a reduced efficiency as compared to non-differentiated counterparts. We propose a model in which activation of the differentiation program in GBM cells enhances their infiltrative potential and that depending on microenvironmental cues a significant portion of these cells are able to revert back to an undifferentiated state with enhanced tumorigenic potential. Thus, effective therapy should target both GSCs and differentiated offspring and targeting of differentiation-associated pathways may offer therapeutic opportunities to reduce invasive growth of GBM.

## Introduction

Glioblastoma (GBM) is an extremely aggressive and clinically difficult to treat cancer that is in part caused by its highly invasive nature [1, 2]. In GBM, tumor initiating stem cells or cancer stem cells (CSCs) have been identified and are commonly referred to as glioblastoma stem cells (GSCs) [3–5]. *In vitro* GSCs are known to be enriched in spherical floating structures, named neurospheres, when cultured in serum-free medium containing bFGF and EGF, which maintains these cells in a largely stem cell or undifferentiated state [6–8]. GSCs are characterized by enhanced tumor initiation potential in comparison to non-GSCs that can be preclinically determined by neurosphere formation and tumor growth potential in immunocompromised mice [4]. Like normal neuronal stem cells (NSCs), which can differentiate into neurons, astrocytes and oligodendrocytes [9, 10], GSCs can also differentiate into similar cell lineages [11]. GSCs have been shown to be highly resistant to chemo- and radiotherapy indicating that these cells may be responsible for tumor relapse after therapy [12, 13].

The highly invasive growth pattern of GBM into the normal brain parenchyma limits the efficacy of surgical intervention leading to the poor prognosis of patients diagnosed with GBM. Nonetheless, surgical debulking in combination with chemo-radio therapy remains the mainstay treatment strategy for GBM [14, 15]. The invasive and diffuse growth pattern of malignant gliomas was recognized by neurosurgeons decades ago; super-radical resections using hemispherectomies even failed to eradicate the tumor cells and led to relapse and formation of secondary lesions in the other hemisphere [16, 17]. Several studies have indicated enhanced invasive potential of GSCs and their involvement in relapse of GBM [18–20]. It is also broadly believed that in epithelial cancers CSCs have elevated invasive potential, which might contribute to “metastatic colonization” in distant organs leading to cancer-related mortality [21, 22]. As CSCs possess tumor-initiating capacity, which is mandatory for the establishment of secondary tumor in distant organs, it is compelling to argue that CSCs are more invasive in nature.

In the current study we addressed the question whether undifferentiated GBM neurosphere-cultured cells have elevated invasive potential when compared to serum-differentiated counterparts using in vitro and in vivo assays. In addition, the involvement of Matrix metalloproteinase-9 (MMP9) in tumor invasion was examined. We propose a model in which early differentiated GBM cells are most invasive and depending on cues of the microenvironment are able to revert back to a stem cell state facilitating tumor propagation.

## Materials and Methods

The primary material used in this study was surgical leftovers obtained from anonymous GBM patients. The material was obtained after approval and following the ethical guidelines of the Medical Ethics Review Committee (METC) of the University Medical Center Groningen (UMCG).The animal experiments described in this manuscript were approved by the Animal Ethical Committee (DEC) and conducted in compliance with the Animal Welfare Act Regulations. Care was taken at every step to minimize suffering to the animals by the correct administration of anesthesia and analgesic agents whenever needed. Further the animals were monitored daily by the researcher (JJ). The animal welfare officer of the Central Animal Facility (CDP), UMCG also monitored the animals twice a week.

### Cell culture and treatments

GG1, GG9, GG12, GG14 and GG16 cells were newly generated from left over GBM primary material under approval and following the ethical guidelines of the Institutional Review Board of the UMCG and as described elsewhere [23]. The cells were found to represent different molecular subtypes, GG1 and GG16 mesenchymal (MES) and GG9, GG12 and GG14 proneural (PN). Cells were propagated as neurospheres in neural stem cell medium (NSM) which is composed of-Neurobasal A-Medium (Gibco Life Technologies, Bleiswijk, The Netherlands) supplemented with 2% B27 supplement (Gibco Life Technologies), 20ng/ml EGF (R&D systems, Abingdon, UK), 20 ng/ml bFGF (Merck-Millipore, Billerica, MA, USA), 1% pen/strep and 1% L-glutamine (Gibco Life Technologies). The GBM cell line GSC23 (PN) was a kind gift from dr. H. Colman (University of Utah, Salt Lake City, USA) and was described previously [24]. Neurospheres were differentiated by first pelleting and washing cells with PBS followed by accutase (Sigma-Aldrich, St Louis, MO) treatment and repeated pipetting in NSM medium to dissociate cells. The single cell suspension was seeded on poly-L-lysine- (P8920, Sigma-Aldrich) or laminin-coated plates (L4544, Sigma-Aldrich) in differentiation medium containing- Neurobasal-A medium supplemented with 10% foetal calf serum, 2 mM L-glutamine, 100 units/mL penicillin and 100 μg/ml streptomycin (Gibco Life Technologies) for 10 days, with media changes every 2–3 days.

Cells were maintained at 37°C in a humidified atmosphere with 5% CO_2_. When indicated, cells were treated with small molecule inhibitor of MMPs, CP 471474 (Axon Medchem, Groningen, The Netherlands). The inhibitor was added at a concentration of 50 and 100 nM 24 hrs prior to using the cells for experiments.

### Immunofluorescence microscopy

Neurospheres were dissociated and seeded on poly-L-lysine-coated coverslips and allowed to differentiate for 10 days in medium supplemented with 10% FCS with a medium change every 3 days. Whole differentiating neurospheres were seeded on poly-L-lysine-coated coverslips in medium supplemented with 10% FCS and after 5 days cells were fixed using 4% para-formaldehyde for 10 minutes at room temperature. After another PBS wash the cells were permeabilized with ice-cold 0.1% Triton X-100 (X100, Sigma-Aldrich) in PBS for 10 minutes at room temperature, washed again and incubated in PBS containing 2% normal goat serum (X0907, Dako, Glostrup, Denmark), 2% BSA (PAA Laboratories GmbH, Colbe, Germany) and 1% Tween 20 (P2287, Sigma-Aldrich) for 1 h at room temperature. Cells were stained for 1.5 h at room temperature with primary antibodies against β III-Tubulin (ab76287, Abcam, Cambridge, UK), GFAP (Z0334, Dako), SOX2 (4900, Cell Signaling Technology, Danvers, MA). Subsequently, cells were washed with PBS and incubated with secondary antibodies goat anti-rabbit Cy3 (AP132C, Merck Millipore) or goat anti-mouse Alexa Fluor 488 (A-21121, Thermo Fisher Scientific, Waltham, MA) at a concentration of 1:200 for 1 h at room temperature.

Hoechst (Sigma-Aldrich H6024) staining was performed for 5 minutes followed by mounting the coverslips with Kaisers glycerin (Merck Milipore). Cells were examined by fluorescent microscopy (Leica DM6000, Leica Microsystems GmbH, Mannheim, Germany) and images were captured using Leica DFC360 FX camera.

### Wound healing assay

Six-well plates were coated with 1:10 diluted poly-L-lysine and 0.5 μg/ml laminin (L4544, Sigma-Aldrich). Cells were most suitable for use in this assay since the adhere seeded at a density of 2x10^5^ cells/well. Neurospheres were dissociated and single cells were grown in NSM medium until a monolayer was formed. For the differentiated condition, cells were grown in differentiation medium for 10 days, with medium changes every 2–3 days, until a monolayer was formed. A scratch was made in the monolayer by manually scraping with a 10 μl pipette tip and the medium was aspirated to remove detached cells. GSC23 cells remained well attached to the plates after scratching making them most suitable for use in this assay. The cells were then incubated with medium supplemented only with 10% FCS for 24 h. Images were acquired with a Leica camera mounted on an inverted microscope and were processed using image J software. The distance cells migrated was determined by measuring the wound area after 24 h corrected for the wound area at time 0 h. The values obtained were expressed as a migration percentage, setting the gap width at 0 h as 0%.

### Limiting dilution assay

Undifferentiated and differentiated cells were sorted based on the forward and sideward scatter pattern using a flow cytometer (BD Biosciences, San Jose, CA). Single cells were seeded in 96-well plates at a density of 10, 20 or 40 cells/well in a volume of 150 μl NSM; per cell density, 3 plates was used and cells were replenished with 75μl of NSM every 5 days. After 3 weeks, the number of neurospheres per well were counted.

### Western blotting

Western blotting was performed as described previously [23]. Membranes were probed overnight at 4°C with the following primary antibodies: β III Tubulin (ab76287, Abcam), GFAP (Z0334, Dako), SOX2 (4900, Cell Signaling), Nestin (sc-23927, Santa Cruz Biotechnology INC), MMP9 (ab76003, Abcam), Rabbit-α-mouse (1:1500; P0260, Dako) and goat-α-rabbit (1:1500; P0448, Dako). HRP-conjugated secondary antibodies were used for detection using Lumi-Light^PLUS^ Western Blotting Substrate (12015196001, Roche Life Sciences, Branford, CT). Membranes were probed with β-actin antibody (0869100, MP Biomedicals, Santa Ana, CA) to confirm equal loading. MMP9 bands were quantified by densitometry and their intensities were corrected for the corresponding β-actin bands; the intensity in neurospheres was set at 1.

### Transwell invasion assay

The invasion potential was determined on collagen-coated Transwell inserts with 8μm pore size (Becton Dickinson B.V., Breda, The Netherlands). GG16 cells proliferated fast and maintained some proliferative properties after differentiation and were used in this assay. Cells were trypsinized and resuspended in 0.1% FCS containing medium. 150μl of a cell suspension containing 5 x 10^4^ cells were added to the Transwell in triplicates per condition. 10% FCS or 0.1% FCS was added to the lower wells as chemoattractants. Cells that migrated/ invaded and appeared on the bottom surface of the Transwell insert membrane were fixed with 75% methanol/25% acidic acid for 20 min and stained with 0.25% Coomassie blue in 45% methanol/10% acetic acid followed by washing with demi water. The membranes were subsequently cut out and mounted on microscopic slides for quantification. Representative pictures of the membranes with cells were acquired at 5x magnification and the total number of cells on fifty individual fields per membrane was counted; average numbers and standard deviation of invading cells for every condition were calculated.

### Intracranial Injection mouse model

GG16 neurospheres (GG16 Nsp) and differentiated (GG16 Dif.) cells were prepared for intracranial injection in NOD scid gamma mice (NOD.Cg-*Prkdc* ^*scid*^ *Il2rg* ^*tm1Wjl*^/SzJ)/ NSG mice (inbred strains obtained from Central Animal Facility, Groningen). 3x10^5^ GG16 Nsp and GG16 Dif. cells were injected in the striatum of the animals (6 mice for each condition) using a stereotactic frame to determine tumorigenicity and invasive growth. Mice were monitored on a regular basis. At day 7 and day 21 post implantation of the cells the animals were perfused under anesthesia with PBS and 4% paraformaldehyde as described earlier [25]. The brains were harvested and fixed in 4% para-formaldehyde for 24 hrs at 4°C and embedded in paraffin and prepared for IHC. These experiments were approved by the committee for Animal care, and conducted in compliance with the Animal Welfare Act Regulations.

For quantifying tumor invasion, from each mouse brain 4 to 6 serial sections were evaluated for infiltrating tumor cells after immunohistochemical staining for Nestin that specifically stains the implanted tumor cells. A cell was considered infiltrating when (1) it was positioned outside the core or the original implantation site with preexistent mouse brain cells lying in between the core and the infiltrating cells and (2) Nestin staining was present around a clearly visible nucleus.

### Quantitative real-time polymerase chain reactions (RT-PCR)

Total RNA was isolated from either neurospheres or differentiated GBM cells using RNeasy mini kit (Qiagen, Hilden, Germany) as per the instruction of the manufacturer. 1μg of total RNA was used to reverse transcribe into cDNA by a RNase H+ reverse transcriptase using iScript cDNA synthesis kit (BioRad, Hercules, CA) as per manufacturer’s instructions. The cDNAs were stored at -20°C until used for quantitative RT-PCR. Real-time (RT)-PCR was performed in an ABI PRISM 7900HT Sequence Detector (Applied Biosystems, Foster City, CA) with the iTaq SYBR Green Supermix with Rox dye (Biorad) and amplification was performed with the following cycling conditions: 5 min at 95°C, and 40 two-step cycles of 15 sec at 95°C and 25 sec at 60°C. The reactions were analyzed by SDS software (Version 2.4, Applied Biosystems, Foster City, CA). The threshold cycles (Ct) were calculated and relative gene expression was analyzed after normalizing for GAPDH, house-keeping gene. Human primers used are listed in S1 Table. For meta-gene analysis, fold induction were log transformed and data was imported to Genesis software (Graz University of Technology, Graz, Austria (genome.tugraz.at/genesis)) for performing clustering and heatmap visualization.

### Immunohistochemistry

Coronal sections of 3 μm thickness were prepared from mouse brains injected with GBM cells. The sections were dried overnight at 55°C. Prior to staining, tissues were deparaffinized in xylol and rehydrated in graded series of ethanol. Antigen retrieval was required for Ki-67, MMP-9 and Nestin through microwave pretreatment with Tris/EDTA, Tris/HCl or citrate buffer, respectively. Subsequently endogenous peroxidase was blocked for 30 minutes using 0.3% H_2_O_2_ at room termperature (RT). Then slides were incubated with primary antibodies diluted in 1% BSA/PBS for 1 hour at RT. Antibodies used were GFAP (Z0334, Dako) diluted 1:250, Ki-67 (MIB-1, Dako) diluted 1:300, MMP-9 (C-20, Santa Cruz Biotechnology INC) diluted 1:50, and Nestin (10C2, Santa Cruz Biotechnology INC) diluted 1:100. Secondary and tertiary antibodies were diluted in 1% BSA/PBS and supplemented with 1% AB-serum and incubated for 30 minutes at RT. Staining was visualized by 3,3’-diaminobenzidine incubation for 10 minutes and sections were counterstained with haematoxylin and mounted. Images were acquired using a C9600 NanoZoomer (Hamamatsu Photonics KK, Hamamatsu City, Japan).

### Statistical Analysis

In-vitro data were represented as the mean ± standard error of the mean (SEM) using the GraphPad Prism version 5.01 (GraphPad for Science, San Diego, CA). Two tail unpaired student’s t-test was used for calculating statistical significance between two groups was unless otherwise mentioned in the figure legends. p values < 0.05 were assumed as statistically significant for all the tests. Differences in the number of infiltrating cells in mouse brain slices of animals implanted with neurospheres or differentiated cells were calculated by Mann-Whitney tests.

## Results

### Serum-induced differentiation of GBM cells

**Fig 1:**
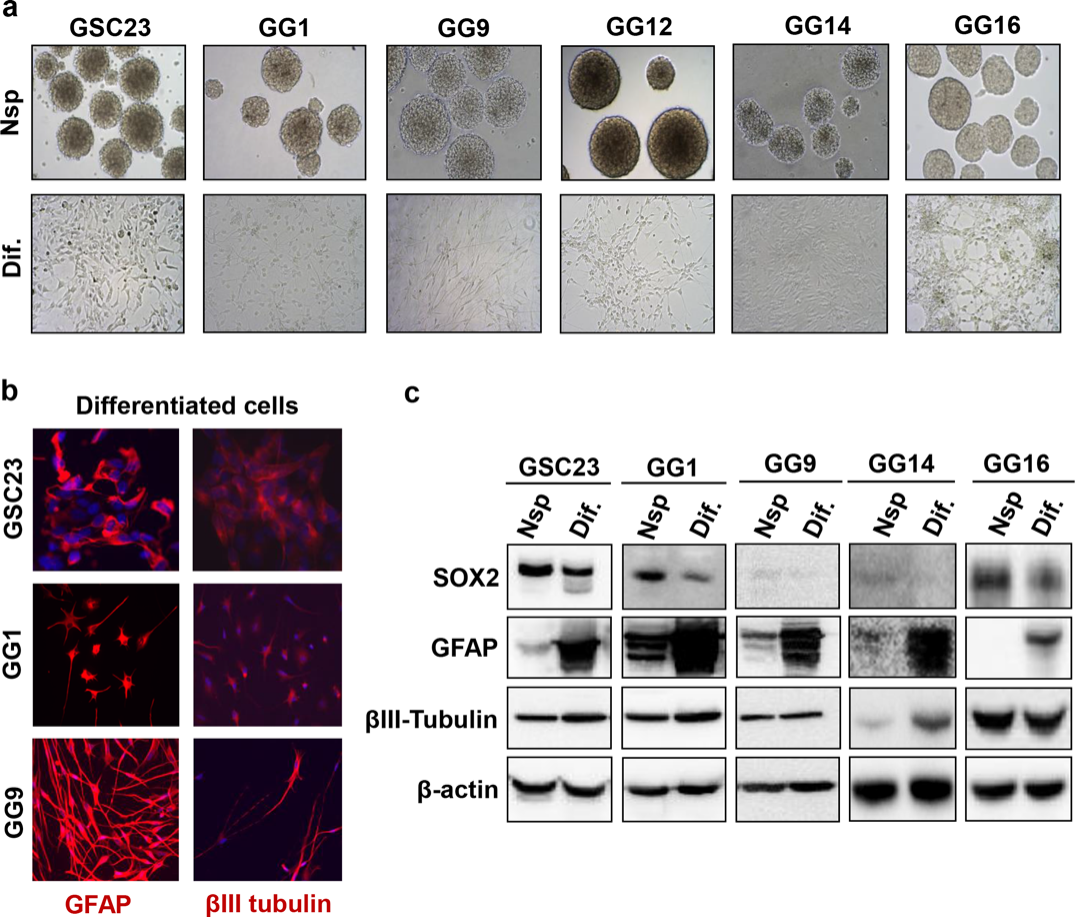
Serum induces mainly astrocytic differentiation in neurospheres. **a.** Pictures of neurosphere cultured and differentiated adherent monolayers of six different cell lines, GSC23, GG1, GG9, GG12, GG14, GG16 (magnification x10). **b.** Immunostaining for astrocytic (GFAP) and neuronal (β3 Tubulin) differentiation markers in differentiated GSC23, GG1 and GG9 (magnification x20; GFAP and β3 Tubulin in red and DAPI in blue). **c.** Western blot detecting the expression of stem cell marker SOX2 and differentiation markers GFAP and β3 Tubulin in differentiated and undifferentiated GSC23, GG1, GG9, GG14 and GG16.

GSC23, GG1, GG9, GG12, GG14 and GG16 cell lines were propagated as neurospheres or exposed to 10% FCS for 10 days to induce differentiation, similar as described by Singh et al [3]. Upon differentiation the cells adhered and acquired mostly a typical star shaped glial morphology (Fig 1A). To further confirm differentiation, cells were stained for the presence of astrocytic GFAP and neuronal β3 Tubulin markers using immunofluorescence microscopy. Both lineage specific markers were detected, though at varying intensity and with GFAP expression being mostly enhanced after FCS-induced differentiation and GG14 cells only showing significant elevation in β3 Tubulin expression upon differentiation (Fig 1B and 1C). On the other hand, the expression of the stem cell marker SOX2 overall appeared to decrease upon differentiation, although to variable extends (Fig 1C).

### Serum-induced differentiation enhances migration and invasion in vitro

**Fig 2:**
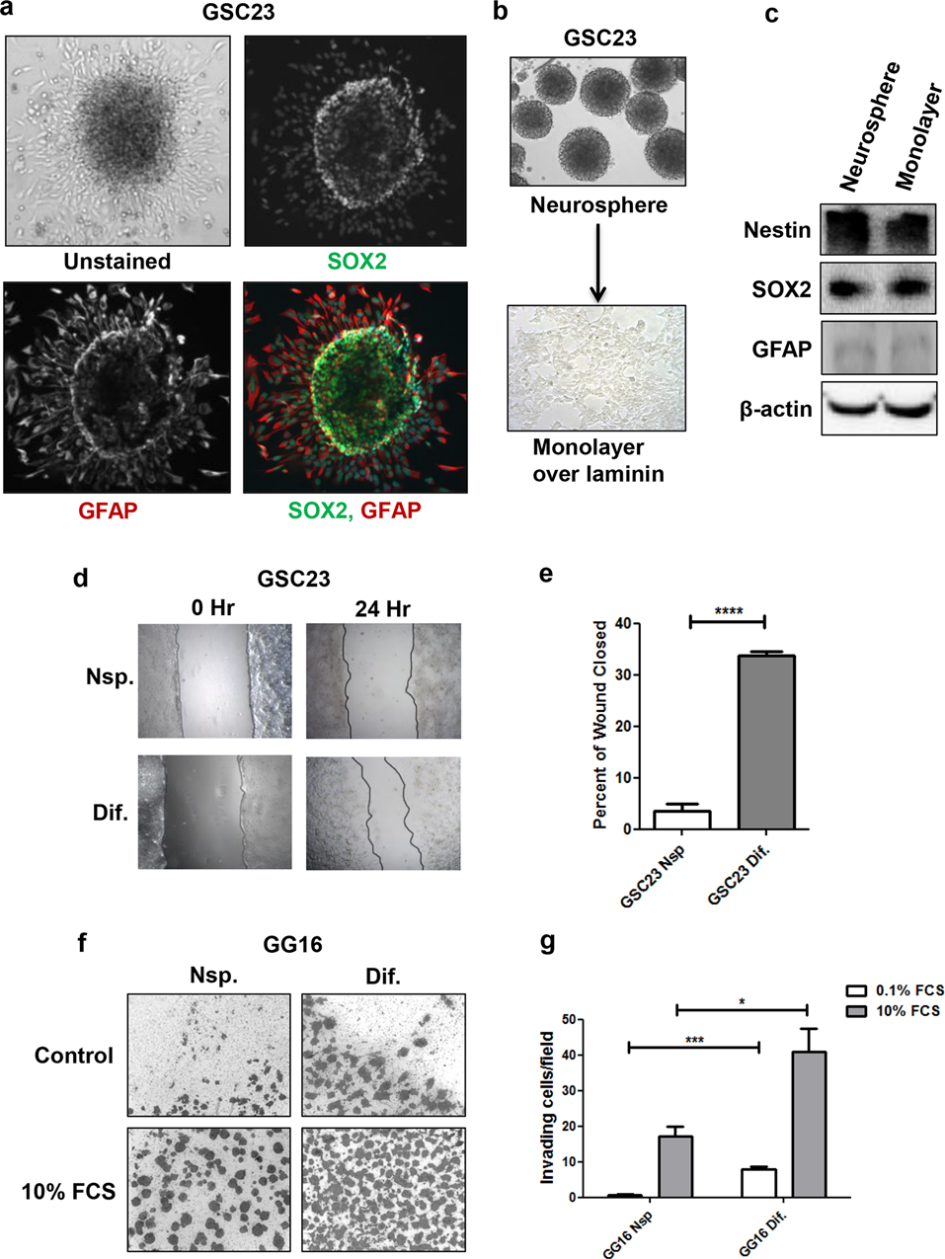
Serum-induced differentiation enhances migration and invasion in vitro. **a.** GSC23 whole neurosphere differentiation on poly-l-lysine coated cover slips in media containing serum, along with co-immunostaining with SOX2 (green) and GFAP (red; DAPI in blue; magnification x10). **b.** Inverted microscope images of GSC23 neurospheres and undifferentiated monolayer GSC23 cells cultured in serum free medium on laminin-coated plates (magnification x10). **c.** Western blot showing the expression of Nestin and the stem cell marker SOX2 and differentiation marker GFAP in the neurospheres and undifferentiated laminin-coated GSC23 monolayer cells. **d.** Wound healing assay performed with differentiated monolayer and undifferentiated laminin-coated monolayer GSC23 cells showing differences in wound closure capacity (magnification x5), quantification of wound closure of 3 independent experiments are shown in (**e**) (**** p<0.0001). **f.** Representative pictures (magnification x5) of transwell membranes comparing the invading capacity of differentiated and undifferentiated GG16 cells; quantification shown in **g.** The bars represent the mean of three independent experiments measured in triplicate ± SEM (*P < 0.05, ***P < 0.001).

When whole GSC23 neurospheres were seeded on poly-L-lysine-coated coverslips and subsequently exposed to 10% FCS for 5 days, we noted that adherent cells started radiating out of the neurospheres (Fig 2A). Upon immunofluorescent staining for SOX2 and GFAP, interestingly, we observed that SOX2-expressing cells appear to be more confined to the core structure of the neurospheres, while the cells that migrated out of the spheres lost SOX2 expression and gained that of GFAP. This suggested to us that serum-differentiated GSC23 cells have potent migratory properties that may possibly exceed that of undifferentiated cells.

To further compare the migratory potential of undifferentiated and serum-differentiated GBM cells wound healing assays were performed. For this, monolayer GBM cell cultures in serum-free medium were established on laminin-coated dishes that previously were shown to retain GSC characteristics [26]. Indeed, the expression patterns of Nestin and the GSC associated marker SOX2 and the differentiation marker GFAP were similar between laminin- and neurospheres cultured GSC23 cells, reflecting their undifferentiated states (Fig 2B and 2C). Wound closure was monitored after 24 hours and showed that differentiated GSC23 cells have an approximately 8-fold higher migratory capacity than the undifferentiated counterparts (Fig 2D and 2E). Similarly, collagen-coated transwell inserts were used to evaluate the invasive potential of differentiated and undifferentiated GG16 cells (Fig 2F and 2G). GG16 differentiated cells showed already an increase in basal invasion capacity of ~ 6 fold compared to the undifferentiated cells, which was strongly enhanced upon addition of 10% FCS as chemoattractant and differentiated cells displayed more than 2-fold higher level of invading cells. In summary it is evident that in an in vitro setting serum-differentiated cell are more migratory and invasive than undifferentiated cells.

### Serum-differentiated GBM cells show enhanced infiltrating potential in vivo

**Fig 3:**
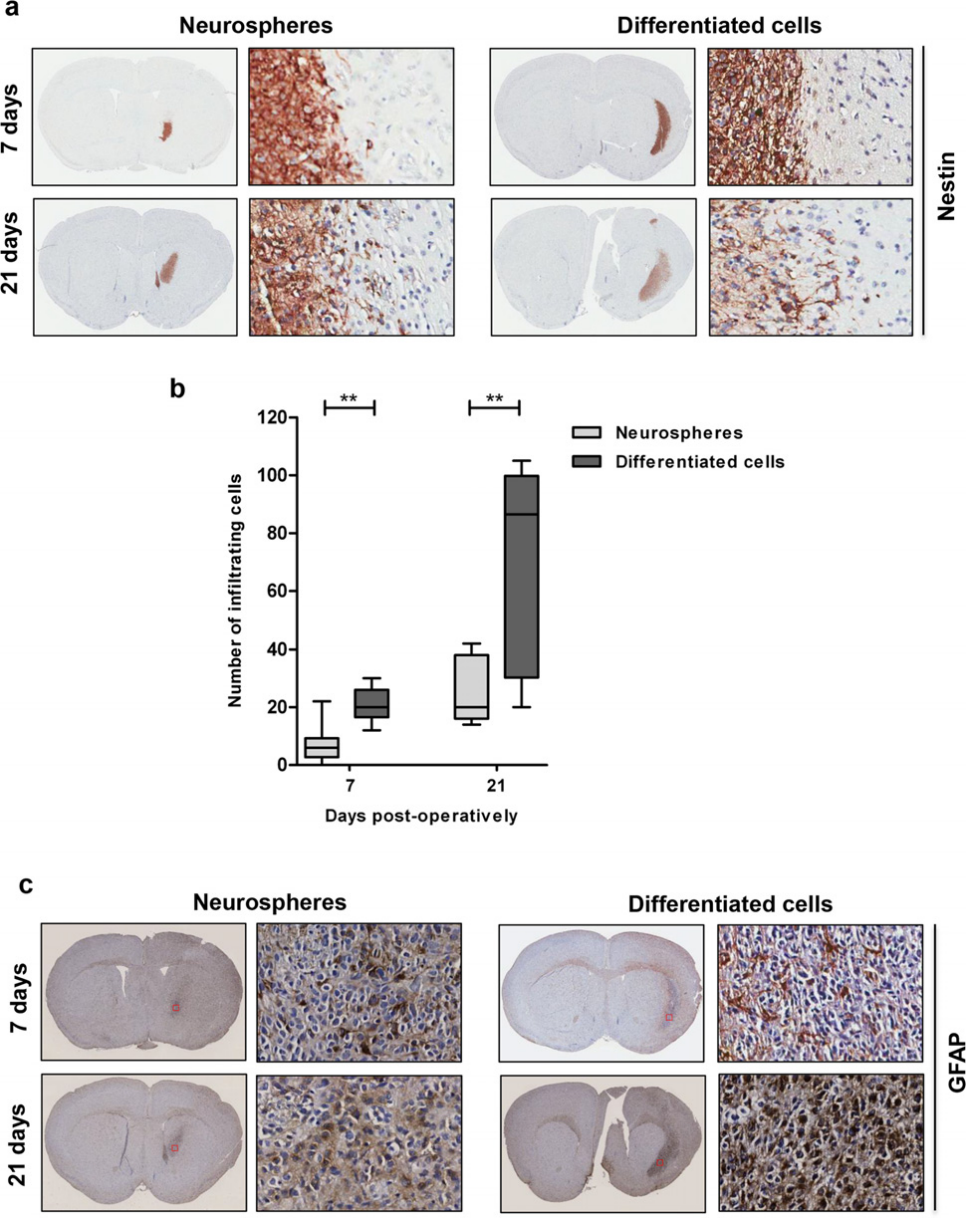
Serum-differentiated GBM cells show enhanced infiltration in vivo. **a.** Immunohistochemical staining with Nestin on xenografts derived from differentiated (GG16D) and undifferentiated (GG16 Nsp) at day 7 and day 21 post intracranial injection; overview (magnification x5) and enlarged picture (magnification x20) are being shown. **b.** Mann-Whitney U tests showing significant differences in the number of infiltrating cells between implanted neurosphere and differentiated cells, both at 7 days (p = 0,001) and 21 days (p = 0,004). The average number of infiltrating cells per section and standard deviations are indicated. **c.** Immunohistochemical staining with GFAP.

Next, we compared the behavior of tumors originating from either differentiated or undifferentiated GBM cells upon intracranial implantation. For this serum-differentiated and undifferentiated GG16 cells were injected into the striatum of NSG mice (n = 6 per condition) and at day 7 (n = 3) and day 21 (n = 3) post-injection the animals were sacrificed and brains harvested for immunohistochemical analysis. Nestin staining allowed the comparison of tumor cell infiltration (Fig 3A and 3B). Quantification of the number of infiltrating cells showed a time-dependent increase in infiltrating cells. However, the number of infiltrating cells is larger in the serum-differentiated group compared with the neurospheres group. The median cell numbers infiltrating in the neurosphere group at 7 days was 6, and at 21 days 20; in the differentiated group at 7 days was 20, and at 21 days 86,5 (Fig 3B). There was no significant difference in proliferative potential as indicated by similar Ki67 staining patterns between the tumors derived from differentiated and undifferentiated cells (S1 Fig). At day 7 post-injection most of the tumor cells were largely negative for GFAP in both groups with few cells showing positive staining. However, at day 21 the GFAP levels in the tumor increased in both groups, with particularly 1 out of the 3 animals in the differentiated group showed a strong increase in GFAP levels (Fig 3C). Also note that the expression of GFAP in GG16 cells was relatively low compared to the other GBM cells in vitro and that the brain microenvironment apparently also induces GFAP expression, i.e. differentiation (Fig 1C) and may explain the relative low expression of GFAP in the GG16 xenografts. Overall we obtained evidence that serum-differentiated GG16 cells have elevated invasive potential in comparison to the undifferentiated cells in the intracranial mouse model.

### Serum-differentiation leads to enhanced MMP9 expression and chemical inhibition of MMP9 reduces invasion in vitro

**Fig 4:**
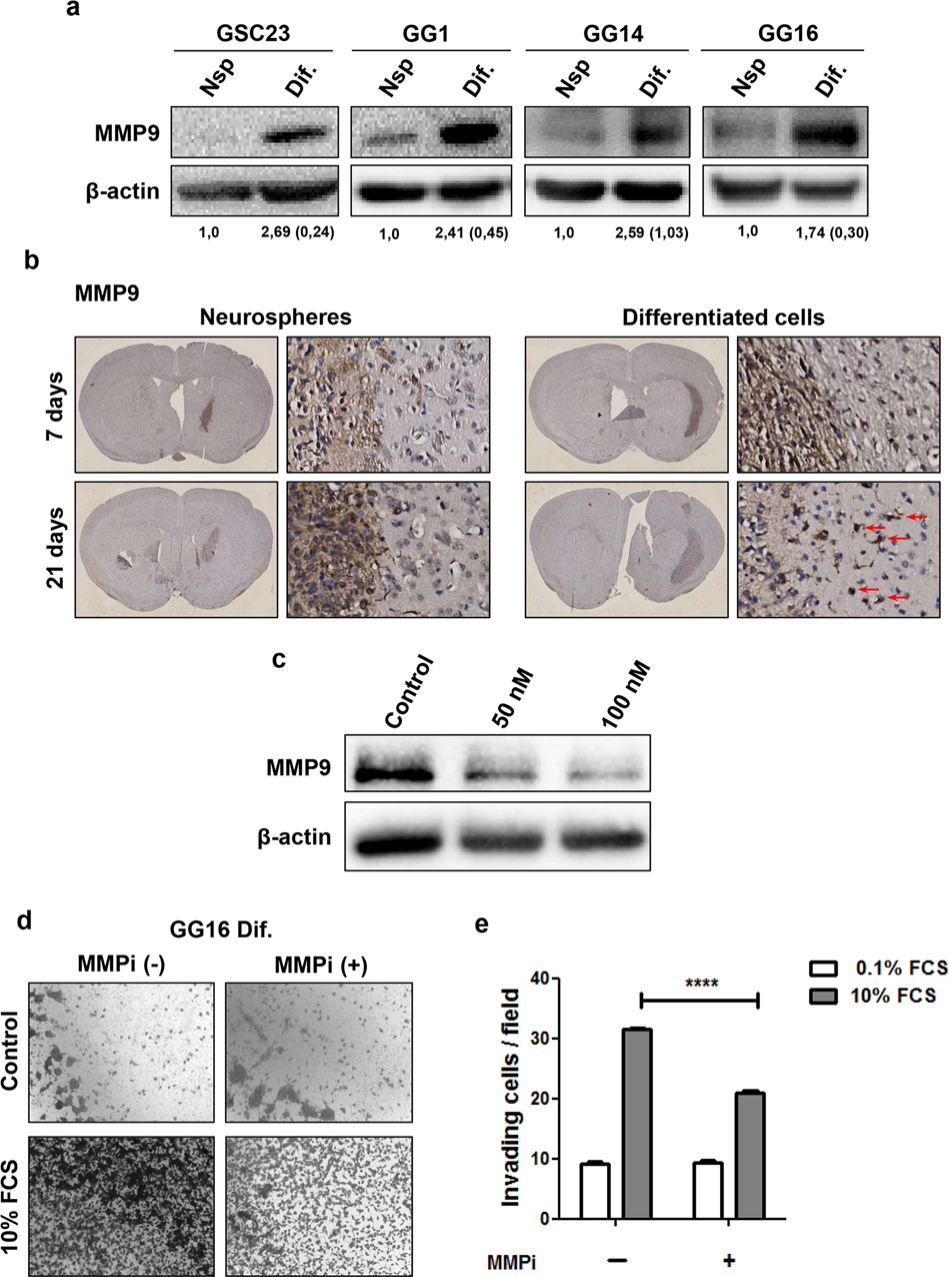
Serum-differentiation leads to enhanced MMP9 expression. **a.** Western blot showing MMP9 expression in differentiated and undifferentiated GSC23, GG1, GSC14 and GG16 cells. The numbers (SD between parentheses) indicate the intensity of the quantified bands corrected for loading (β-actin) and the level in neurospheres was set at 1. **b.** Immunohistochemical staining for MMP9 on xenografts derived from differentiated and undifferentiated GG16 at day 7 and day 21 post intracranial injection showing elevated MMP9 expression in xenografts derived from the differentiated cells in comparison to the undifferentiated cells; overview (magnification x5) and enlarged picture (magnification x20). **c.** Western blot showing a decrease in MMP9 expression in differentiated GG16 cells following the administration of CP 471474, a chemical inhibitor of MMP9. **d.** Representative pictures (magnification x5) of transwell membranes showing a reduction of the invading capacity of differentiated GG16 cells following pre-treatment with the MMP9 inhibitor; quantification is shown in **e.** Data represent the means ± SEM of 3 independent experiments where 30 neurospheres were included in each experiment (****P < 0.0001).

MMP9 belongs to a large family of proteases that play important roles in degrading the extracellular matrix thereby facilitating tumor invasion [27]. Also in GBM an important role for MMP9 has been reported in mediating invasion and migration [28, 29]. Therefore, we studied the possible involvement of MMP9 in our models, GG1, GG16, GG14 and GSC23. We observed that FCS-differentiated cells expressed a significantly higher level of MMP9 compared to the undifferentiated cells (Fig 4A; quantified data are depicted in S2 Fig). Overall little or no elevation in the level of MMP2 was observed following differentiation in none of the cell lines used in this study (S3 Fig). In parallel, immunohistochemical staining for MMP9 on the intracranial tumors also showed elevated MMP9 expression in xenografts derived from FCS-differentiated GG16 cells; particularly MMP9 expression appeared higher in tumor cells that were moving out of the main tumor mass and invading the normal brain (Fig 4B). In addition, also note some MMP9 expression in the tumor cores, which may reflect differentiation of cells and is in agreement with the presence of GFAP (see Fig 3B).

In order to further validate the role of MMP9 in mediating the enhanced invasive potential of differentiated cells we used a selective chemical MMP inhibitor (MMPi) (CP471474) known to potently block MMP9 activity with an IC50 value of 13nM [30]. At 50nM and 100nM for 24 h the inhibitor also induced a significant reduction of MMP9 expression at the protein level in the differentiated GG16 cells (Fig 4C). In transwell assays pretreatment of differentiated GG16 cells with CP471474 (100nM) led to a significant reduction of approximately 30% in invasive potential when compared to untreated cells (Fig 4D and 4E). On the other hand, the administration of CP471474 did not significantly reduce the proliferation potential of GG16 cells both in number and size of spheroids formed in a limiting dilution assay (S3 Fig). Together this indicates that differentiation-enhanced invasive capacity is associated with increased expression and activity of MMP9.

### Serum-differentiated GBM cells can partially revert back to an undifferentiated state and reacquire spheroid forming potential

**Fig 5:**
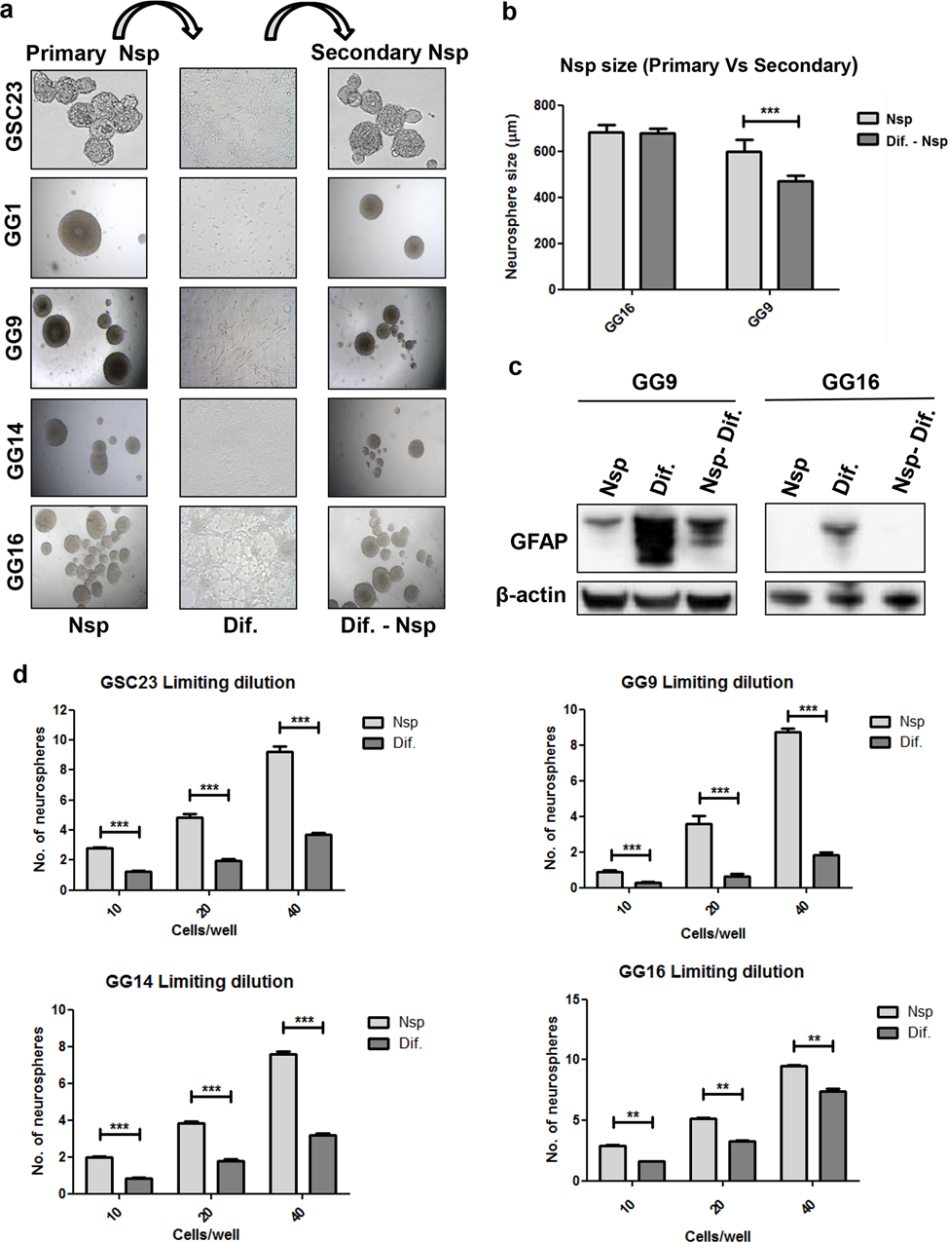
Serum-differentiated GBM cells can revert back to an undifferentiated state and re-acquire stemness. **a.** Serum-differentiated GSC23, GG1, GG9, GG14 and GG16 (primary) neurospheres (Nsp) reverted back to (secondary) neurospheres following exposure to neural stem cell medium (NSM). **b.** Quantification of the size of primary and secondary GG9 and GG16 neurospheres. (***P<0.001). **c.** Western blot showing the expression level of GFAP in undifferentiated (primary Nsp), differentiated and de-differentiated (secondary Nsp) GG9 and GG16 cells. **d.** Limiting dilution assay showing the reduction in the neurosphere formation potential in GSC23, GG9, GG14 and GG16 following differentiation. Data represent the means ± SEM of 3 independent experiments and neurospheres were counted from 96 wells in each experiment (**P<0.01, ***P < 0.001).

Our experiments thus far indicated that serum-differentiated GBM cells have enhanced invasive capacity and appeared at least equally tumorigenic upon intracranial implantation in mice in comparison to the undifferentiated counterparts. Since, according to the CSC model, GBM cells with stem cell characteristics are most tumorigenic, we explored the possibility that differentiated GBM cells may be able to revert back to a stem cell state. Therefore we compared the neurosphere forming potential of undifferentiated and differentiated GSC23, GG1, GG9, GG14 and GG16 cells. Serum-induced differentiated cells, when placed back in serum-free medium started forming neurospheres. These, so called secondary neurospheres, showed in some cases a reduction in size (GG1, GG9, GG14) or were similar to the primary neurospheres (GSC23 and GG16) (Fig 5A and 5B). In secondary neurospheres of GG9 and GG16 the expression level of GFAP was also significantly reduced compared to the differentiated counterparts, similar to levels seen in the primary neurospheres (Fig 5C). Limiting dilution assays demonstrated a significant reduction in neurosphere formation potential of the GSC23, GG9, GG14 and GG16 differentiated cells in comparison to the undifferentiated counterparts (Fig 5D). The number of neurospheres formed was proportional to the number of cells seeded for each cell line. This effect was most pronounced in GG9 cells, showing a reduction of approximately 75% in neurosphere forming capacity of differentiated cells, and was least detectable in GG16 cells with a reduction of around 25 to-50%.

### Dedifferentiation of differentiated GG16 cells leads to decreased invasive potential

**Fig 6:**
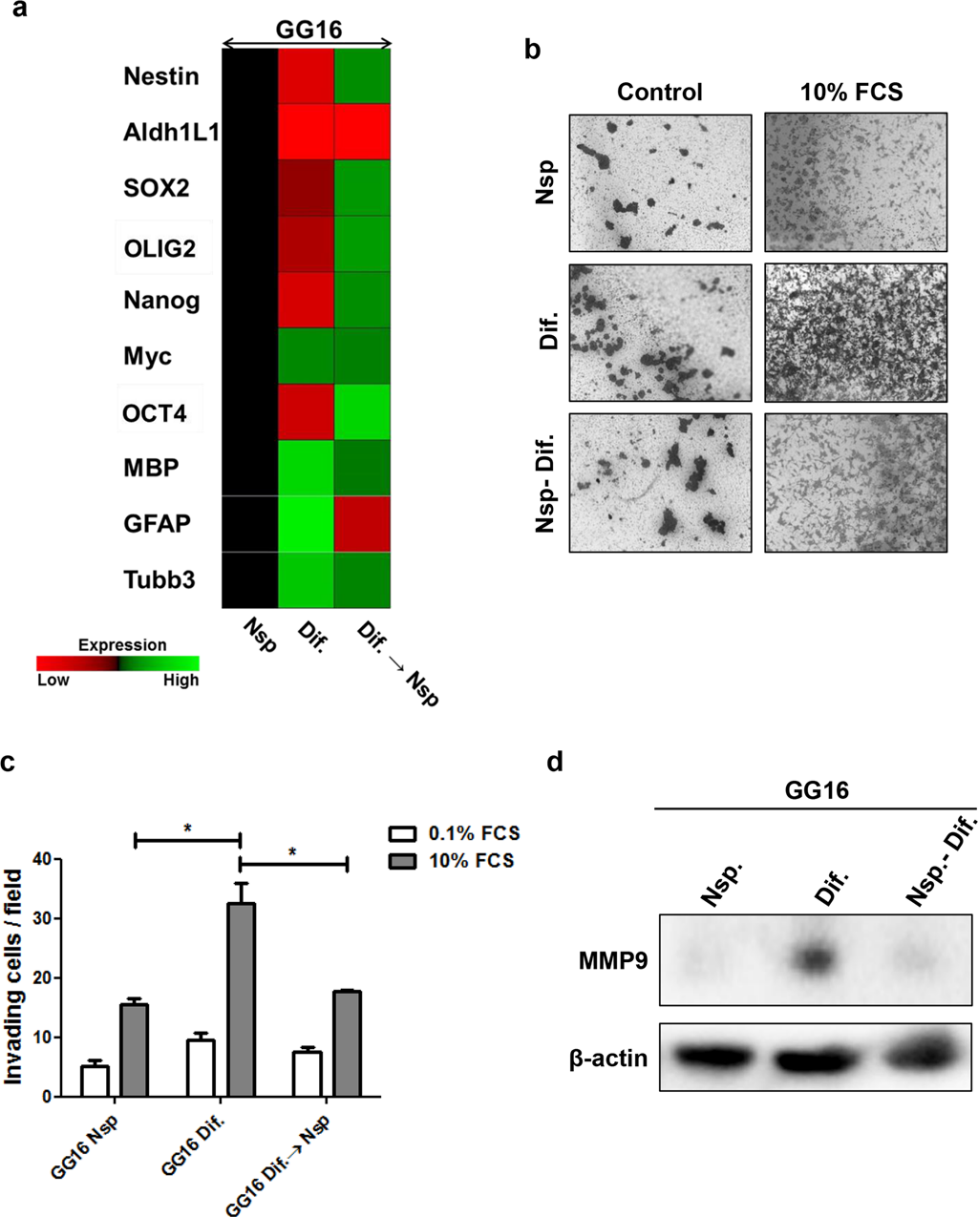
De-differentiation of serum-differentiated GG16 cells reduces their invasive potential. **a.** Heatmap based on fold change of the indicated stem cell and differentiation markers generated by qRT-PCR analyses showing partial gain of stem cell and loss of differentiation markers following dedifferentiation of GG16 cells. **b.** Representative transwell membranes (magnification x5) showing reduced numbers of invading cells after dedifferentiation of GG16 cells (secondary Nsp) that is almost equal to that of primary neurosphere cells; quantification in **c.** showing mean ± SEM of 3 independent experiments (*P<0.*05)*. **d.** Western blot showing the absence of MMP9 expression in primary and secondary GG16 neurosphere cells in comparison to the differentiated GG16 cells.

The differentiation state of primary and secondary GG16 neurospheres and differentiated GG16 cells was confirmed by qRTPCR and showed a loss of stem cell markers in differentiated cells compared to the primary and secondary neurospheres, and a gain of differentiation associated gene expression (Fig 6A). Next, the invasive potential was determined in parallel for GG16 primary and secondary neurospheres and serum-differentiated counterparts. It was observed that cells from primary and secondary neurospheres had equal levels of invasive cells, and their invasive capacity was approximately 50% reduced compared to differentiated GG16 cells (Fig 6B and 6C). Furthermore, the expression of MMP9 was also reduced upon dedifferentiation of GG16 cells (Fig 6D).

## Discussion

The poor prognosis associated with GBM is at least in part due to the high infiltration of GBM cells into the normal brain parenchyma. This makes full surgical resection of the tumor virtually impossible [1, 15]. CSCs in GBM have been associated with several of the aggressive characteristics of GBM such as high tumor vascularization, invasive behavior, chemo- and radio-resistance and relapse of disease after surgery [1, 2, 31]. These GSCs possess self-renewal ability and high tumor-initiating potential and are able to differentiate into bulk tumor cells that are commonly believed to lack tumor forming ability [3, 4, 31]. Partially in contrast with this view, our present study shows in in vitro assays that serum-differentiated GBM cells have higher migratory and invasive capacity than undifferentiated neurosphere cultured counter parts that are enriched for GSCs. Further, the tumors originating from intracranial implanted undifferentiated or serum-differentiated cells also showed differences in tumor invasion, with tumors originating from the serum-treated cells showing elevated invasion along the lateral border at day 7 and 21 post intracranial implantation of the tumor cells. Following serum-differentiation, upregulation of MMP9 was detected in GBM cells and elevated expression of MMP9 was also noticed in the invading tumor cells originating from the serum-differentiated cells in comparison to the tumors that originated from the undifferentiated cells.

The association of elevated expression of MMP9 with enhanced invasive potential in GBM has been previously reported [28, 29, 32, 33]. For tumor cell invasion interactions with the ECM needs to be modified for allowing cells to move into more distant parts of the brain. Degradation of the ECM is known to be facilitated by the secretion of proteolytic enzymes such as the plasminogen activators and the MMPs, and in GBM MMP2 and MMP9 have been connected to invasion [34]. Indeed, we found that the chemical inhibition of MMP9 by MMP inhibitor (MMPi)-CP 471474 in differentiated GG16 cell significantly reduced their invasive potential in vitro, showing that migration and invasion are at least in part linked with the presence of MMP9.

Earlier studies have associated CSCs with a high migratory capacity. For example, the existence of migrating CSCs has been proposed that disseminate by undergoing EMT and at the same time retain stem cell functionality allowing metastatic colony formation [35–37]. The presence of such migrating CSCs with distinct features compared to the regular CSC compartment has not been confirmed as yet in GBM. However, previously it was demonstrated that CD133 positive GSCs have enhanced invasive capacity when compared to the non-GSC CD133 negative cell fractions [19]. On the other hand, Weber et al. reported lower migration capacity of GSCs represented by the side population in Hoechst exclusion assays, when compared to the non-side population [38]. Regardless of the validity of the different CSC markers used and apparent contrasting conclusions of these reports, these studies did not compare the effect of serum-induced differentiation on invasiveness.

Campos et al. reported that CD133 positive GSCs treated with serum and all-trans-retinoic acid reduced their tumorigenicity and invasive behavior, the latter being associated with reduced MMP2 expression [39]. However, contrary to our experiments in which serum-differentiated and undifferentiated neurosphere cells were directly compared for invasive capacity, the authors used pre-formed neurospheres that were exposed to differentiation-inducing medium after which invasion was monitored for long time periods, up to 12 days.

Interestingly, we also observed that the serum-differentiated cells could revert back to an undifferentiated state upon seeding in serum-free medium suggestive of de-differentiation. Indeed this was associated with reduced expression of differentiation markers and gain of stem cell markers. Along with this the serum-differentiated GBM cells were able to form neurospheres, although with a reduced capacity (2- to 4-fold) when compared to the undifferentiated counterparts. Of note, the invasive capacity of the de-differentiated cells (secondary neurospheres) also decreased and became comparable to that of the undifferentiated parental cells (primary neurospheres), and was accompanied by a down-regulation of MMP9 levels.

Although serum is often used as an inducer of differentiation in GBM models in vitro, it should be noted that exposure times likely affect the degree of differentiation. For example, Lee et al. reported differences in characteristics between short and long time period serum exposed GBM neurospheres with respect to their neurosphere forming ability and tumorigenicity [6]. In the current study we used serum-differentiation conditions as earlier described by Singh et al [3], representing shorter exposure times than used by Lee et al. [6] and possibly leading to more early differentiation stages. Regardless of this, our findings indicate that GBM neurosphere cells pushed out of the stem cell state by serum, as evidenced by loss of stem cell marker and gain of differentiation marker expression, have enhanced migration/invasion potential.

**Fig 7:**
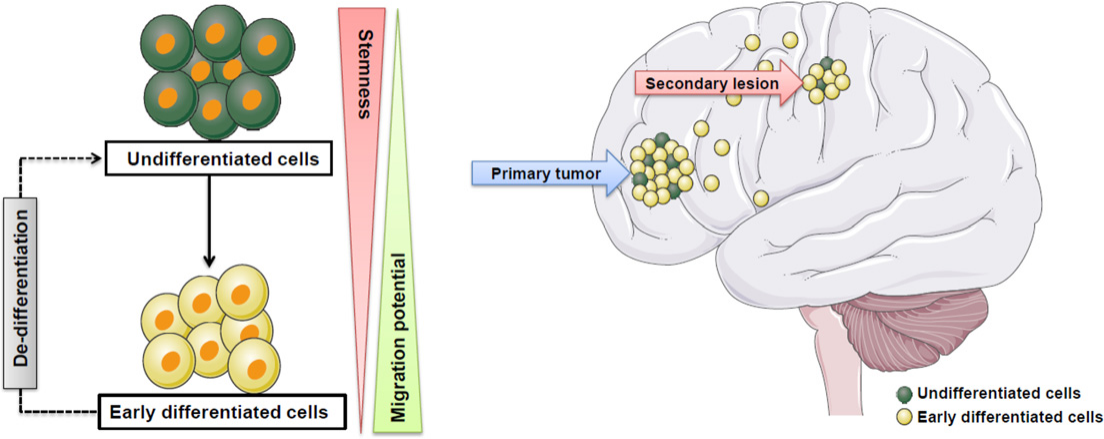
Model depicting the proposed effect of differentiation on GBM progression. **a.** Differentiation enhances the migration and invasive potential of GBM cells. The differentiated cells can (partially) revert back to an undifferentiated/ stem cell state involving signals derived from the microenvironment. **b.** During GBM progression differentiated cells emanating from the primary tumor have enhanced infiltrative capacity and penetrate deeper in to the normal brain parenchyma. The early differentiated cells can revert back to an undifferentiated/stem cell state with unlimited proliferative potential and produce a secondary lesion away from the primary tumor. Alternatively an additional possibility is that the more invasive differentiated GBM cells pave the way for GSCs by generating a passage and suitable conditions for GSCs to follow their track.

Our findings are somewhat reminiscent to the behavior of NSCs that reside in the subventricular zone of the mouse brain. These cells produce transit-amplifying progenitors, which in turn give rise to more differentiated neuroblasts. While the NSCs remain in the subventricular zone these neuroblasts migrate towards the olfactory bulb [31, 40–42]. Thus, it is not the stem cells that migrate through the brain, but the more differentiated neuroblast progenitors. This is in concurrence with a model where GSCs remain at the primary tumor site while upon differentiation cells start to migrate throughout the brain forming secondary lesions (see also Fig 7). It is possible that recurrences that are found further from the primary lesion are the result of more differentiated cells infiltrating deeper in to the brain, and eventually under the influence of microenvironmental cues revert back to a more undifferentiated/ SC state fueling the formation of secondary lesions with unlimited proliferative potential. An analogous de-differentiation process has been detected in astrocytes that were able to revert into NSCs as a result of secretory factors that were produced and released after injuring the spinal cord of adult rats [43, 44]. Thus, similarly it is possible that GSCs after differentiation and migration to another location are able to acquire GSC potential by de-differentiation and to regain tumor initiating potential. This is somewhat reminiscent to an earlier proposed ‘go or grow’ model in which GBM cells can sequentially switch from a proliferation (grow) to an invasion (go) status [45] that is controlled by cues coming from the tumor microenvironment. An alternative or additional possibility is that migratory differentiated GBM cells first infiltrate the brain thus creating a corridor for GSCs and preparing a suitable microenvironment for GSCs to colonize the brain. Finally, the finding that undifferentiated GBM cells/ GSCs have less migratory ability than differentiated cells is perhaps consistent with the recurrence pattern of GBM. Around 80–90% of GBM recurrences occur within 2cm of the primary lesion [1, 46], and may be caused by less migratory GSCs which are known to possess high tumor forming potential [47, 48].

In conclusion, our findings support the view that not only stem-like cells are responsible for the highly invasive phenotype of GBM, but that more differentiated GBM cells with high migratory and invasive potential also contribute to dissemination. Thus, effective therapy should target both GSCs and differentiated progeny in GBM and targeting of differentiation-associated pathways may offer therapeutic opportunities to reduce invasive growth of GBM.

## Supporting Information

S1 Fig Ki67 staining. Immunohistochemical staining for Ki67 on xenografts derived from differentiated and undifferentiated GG16 at day 7 and day 21 post intracranial injection showing similar staining pattern in xenografts derived from the differentiated cells in comparison to the undifferentiated cells; overview (magnification x5) and enlarged picture (magnification x20).
(PDF)

S2 Fig MMP2 expression. Western blot showing no significant difference in the levels of MMP2 following differentiation of neurospheres.
(PDF)

S3 Fig Neurosphere formation. Limiting dilution assay showing no significant reduction in the neurosphere formation potential in GG16 cells following the administration of the MMP inhibitor (MMPi)- CP 471474.
(PDF)

S1 Table List of PCR primers used in the study. (DOCX)
