# Supplementary material for: Serum-Induced Differentiation of Glioblastoma Neurospheres Leads to Enhanced Migration/Invasion Capacity That Is Associated with Increased MMP9
Source: PLoS One. 2015 Dec 23;10(12):e0145393. doi: 10.1371/journal.pone.0145393 (PMC4689519; doi:10.1371/journal.pone.0145393)

Figure S1. Immunohistochemical staining for Ki67 on xenografts derived from differentiated and undifferentiated GG16

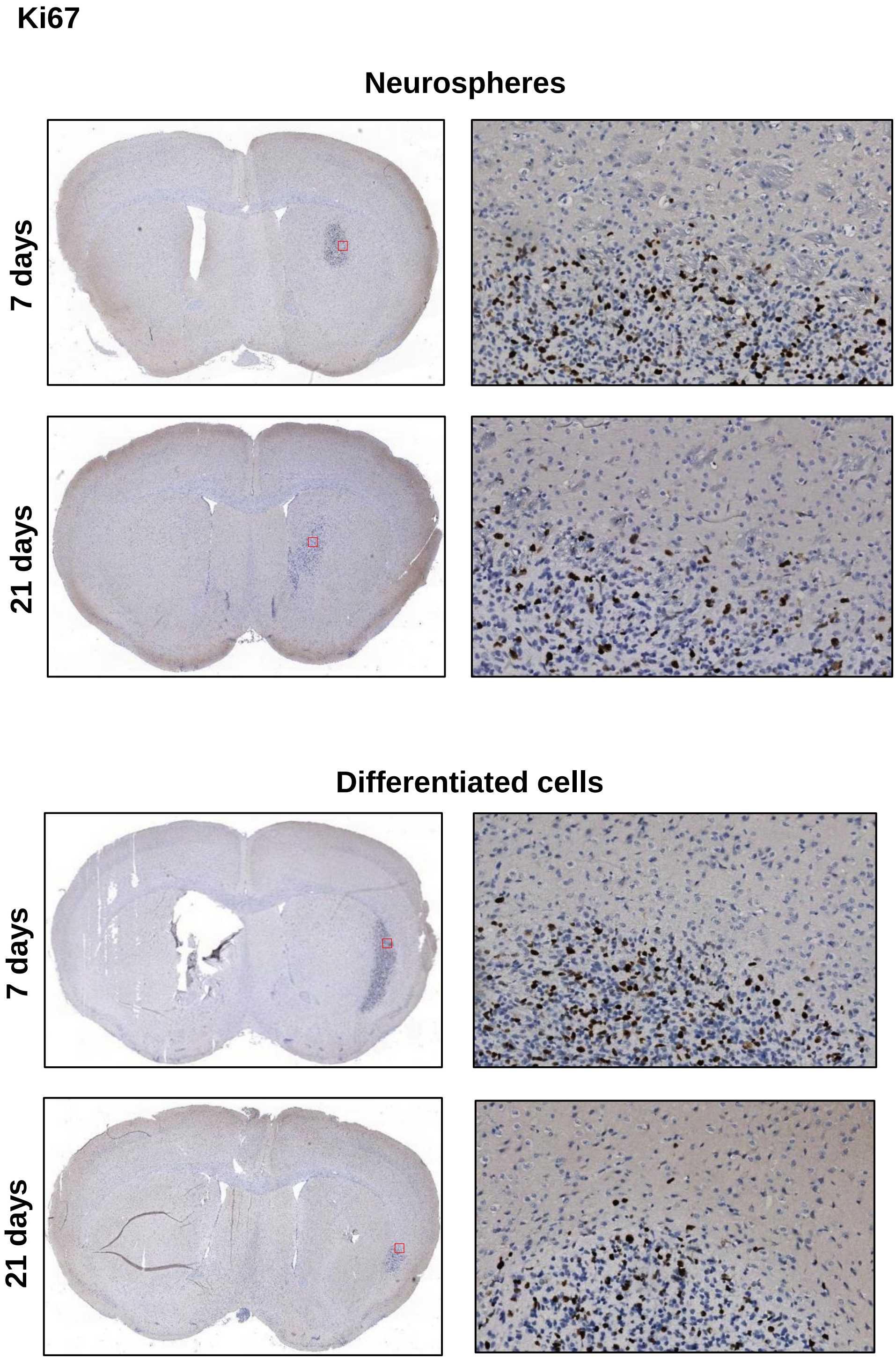

Supplement: S1 Fig — Immunohistochemical staining for Ki67 on xenografts derived from differentiated and undifferentiated GG16 at day 7 and day 21 post intracranial injection showing similar staining pattern in xenografts derived from the differentiated cells in comparison to the undifferentiated cells; overview (magnification x5) and enlarged picture (magnification x20). (PDF) [file pone.0145393.s001.pdf]
