# Supplementary figures and images for: Serum-Induced Differentiation of Glioblastoma Neurospheres Leads to Enhanced Migration/Invasion Capacity That Is Associated with Increased MMP9
Source: PLoS One. 2015 Dec 23;10(12):e0145393. doi: 10.1371/journal.pone.0145393 (PMC4689519; doi:10.1371/journal.pone.0145393)

**Figure S3. Limiting dilution assay using GG16 cells with and without the MMP inhibitor (MMPI)**

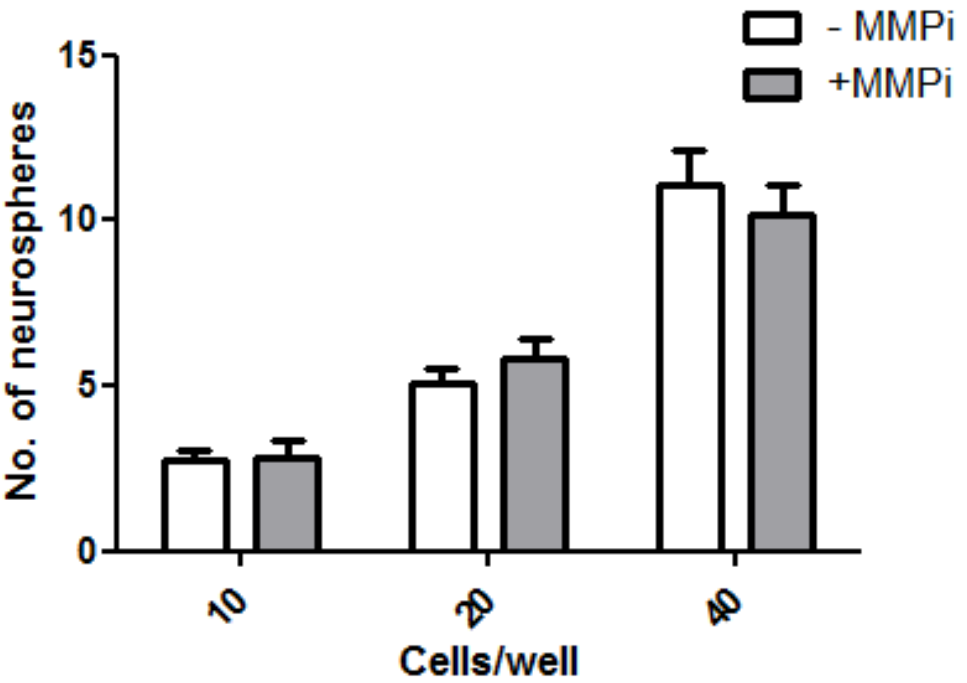

Supplement: S3 Fig — Limiting dilution assay showing no significant reduction in the neurosphere formation potential in GG16 cells following the administration of the MMP inhibitor (MMPi)- CP 471474. (PDF) [file pone.0145393.s003.pdf]
