# Supplementary material for: Serum-Induced Differentiation of Glioblastoma Neurospheres Leads to Enhanced Migration/Invasion Capacity That Is Associated with Increased MMP9
Source: PLoS One. 2015 Dec 23;10(12):e0145393. doi: 10.1371/journal.pone.0145393 (PMC4689519; doi:10.1371/journal.pone.0145393)
Supplement: S1 Table — (DOCX) [file pone.0145393.s004.docx]

**S1 Table.** List of PCR primers used in the study.
