# Supplementary material for: Serum-Induced Differentiation of Glioblastoma Neurospheres Leads to Enhanced Migration/Invasion Capacity That Is Associated with Increased MMP9
Source: PLoS One. 2015 Dec 23;10(12):e0145393. doi: 10.1371/journal.pone.0145393 (PMC4689519; doi:10.1371/journal.pone.0145393)

Figure S2. Western blot showing the similar expression of MMP2 in differentiated and undifferentiated cells

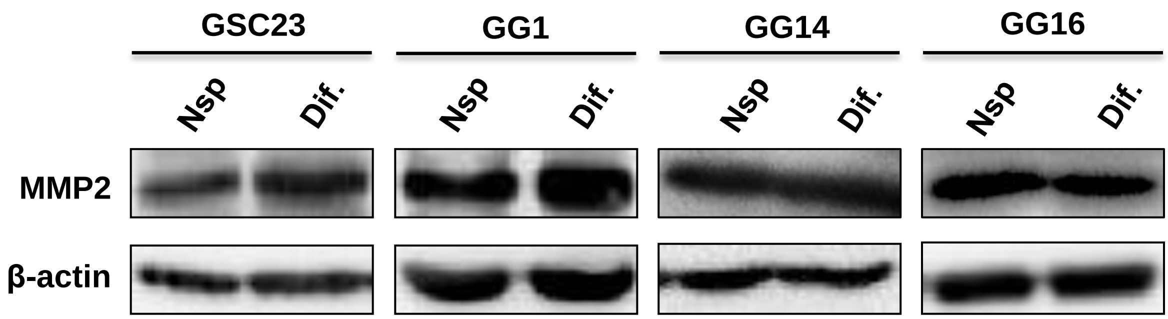

Supplement: S2 Fig — Western blot showing no significant difference in the levels of MMP2 following differentiation of neurospheres. (PDF) [file pone.0145393.s002.pdf]
